# Sexual and reproductive health education and its association with ever use of contraception: a cross-sectional study among women in urban slums, Accra

**DOI:** 10.1186/s12978-021-01322-5

**Published:** 2022-01-15

**Authors:** Abdul-Aziz Seidu, Edward Kwabena Ameyaw, Bright Opoku Ahinkorah, Leonard Baatiema, Samuel Dery, Augustine Ankomah, John Kuumuori Ganle

**Affiliations:** 1 Centre for Gender and Advocacy, Takoradi Technical University, Takoradi, Ghana; 2 College of Public Health, Medical and Veterinary Sciences, James Cook University, Townsville, QLD Australia; 3 School of Public Health, Faculty of Health, University of Technology Sydney, Sydney, NSW Australia; 4 School of Public Health, Faculty of Health, University of Technology Sydney, Sydney, NSW Australia; 5 Department of Health Policy, Planning and Management, School of Public Health, University of Ghana, Accra, Ghana; 6 Department of Biostatistics, School of Public Health, University of Ghana, Accra, Ghana; 7 Population Council, Accra, Ghana; 8 Department of Population, Family and Reproductive Health, School of Public Health, University of Ghana, Legon, P. O. Box LG 13, Accra, Ghana

**Keywords:** Sex education, Contraceptives, Contraceptive use, Women, Urban, Slums, Accra

## Abstract

**Background:**

Sexual and reproductive health education among girls and women has several reproductive health benefits, including improved contraceptive knowledge, contraception use at first intercourse, increased chance of contraceptive use in a lifetime, and effective usage of contraceptives. It is however not clear whether women/girls in urban slums who have had sexual and reproductive health education would likely utilize contraception. This study sets out to test the hypothesis that Accra slum women who have had sex education have higher chances of ever using contraception.

**Methods:**

A cross-sectional survey was conducted among reproductive aged women in two slums (i.e. Agbogbloshie and Old Fadama) in Accra, Ghana. A sample size of 691, made up of respondents who provided responses to the question on ever used contraception, sex education as well as those with complete information on all the other variables of interest was considered in this study. Binary logistic regression models were fitted to examine association between sexual and reproductive health education and ever use of contraception. Crude odds ratios (cOR) and adjusted odds ratios (aOR) at p-value less than 0.05 were used to assess the strength of the association between the outcome and independent variables.

**Results:**

More than half (56.73%) of the women have never received sexual and reproductive health education. Most of the respondents (77.28%) had ever used contraceptives. Women who had no form of sexual and reproductive health education had lower odds of ever using contraception (OR = 0.641, 95% CI 0.443, 0.928) and this persisted after controlling for the effect of demographic factors (AOR = 0.652, 95% CI 0.436, 0.975] compared to those who have ever received any form of sex education. Non-married women as well as women who were exposed to media (newspapers/radio/television) were also more likely to use contraceptives in slums in Accra, Ghana.

**Conclusion:**

The study revealed a relatively low prevalence of sex education among women in urban slums in Accra. However, sex education was found to increase the odds of ever use of contraception. These findings call for intensified sexual and reproductive health education among reproductive aged girls and women in urban slums in Accra using existing informal social networks and local media platforms.

## Plain Language summary

Sexual and reproductive health education among girls and women has several reproductive health benefits, including improved contraceptive knowledge, contraception use at first intercourse, increased chance of contraceptive use in a lifetime, and effective usage of contraceptives. It is however not clear whether women/girls in urban slums who have had some form of sex education would likely utilize contraception more than those who have not. This study sets out to test the hypothesis that Accra slum women who have had sex education have higher chances of ever using contraception. A cross-sectional survey was conducted among reproductive aged women in two slums (i.e. Agbogbloshie and Old Fadama) in Accra, Ghana. A sample size of 691 girls/women aged 15–49 was considered. More than half (56.73%) of the women had never received some form of sex education. Most of the respondents (77.28%) had ever used contraceptives. Women who had no form of sex education had lower odds of ever using contraception and this persisted after controlling for the effect of demographic factors compared to those who have ever received any form of sex education. Non-married women as well as women who were exposed to media (newspapers/radio/television) were also more likely to use contraceptives in slums in Accra, Ghana. In sum, the study revealed that while prevalence of sex education was relatively low among urban slum women, sex education generally increased the chances of ever use of contraception. These findings call for more and targeted sexual and reproductive health education among reproductive aged girls and women in urban slums in Accra using existing informal social networks and local media platforms.

## Background

Unintended pregnancy is one of the major public health issues globally. Around 44% of all pregnancies worldwide are untended, with 74 million in low and middle-income countries [1]. In sub-Saharan Africa, about 29.1% of all pregnancies are unintended, with 35.8% in Ghana [2]. Globally, about 600,000 women die yearly as a result of pregnancy related causes [3]. Almost all (94%) of these deaths occur in low-and middle-income countries including Ghana [3–5]. Use of contraceptives has been identified as an effective approach to fertility regulation by slowing population growth [5–7]. Plethora of research posit that contraceptive use prevents maternal mortality by averting teenage pregnancy, unplanned pregnancies, unsafe abortions, high-risk pregnancies and allowing for spacing of childbearing [7–9]. Equitable access and consistent use of contraception is therefore critical for the achievement of the fifth Sustainable Development Goal (SDG), which aims, among other things, to achieve gender equality and empowerment of all girls and women [10].

High urban population growth in sub-Saharan Africa is largely related to high natural increase, which makes up 75% of the sub-region’s urban population growth [11]. Despite the thought that urban residents may have relatively easy access to health services including modern contraceptives relative to rural residents, evidence indicate that this may be untrue due to the distinct living conditions within cities [12, 13]. In particular, women and girls in informal urban settlements or slums encounter diverse health and socio-economic challenges and may thus experience substantial disadvantages [12–15]. Evidence suggest that the urban poor, including slum dwellers, often tend to have poor reproductive health outcomes as a result of inadequate access to formal health services, unemployment, and overcrowded living conditions [15, 16]. Consequently, unmet need for family planning appears immanent in urban slums [15, 17–19].

Burgeoning evidence, however, suggest that sex education may boost contraceptive use. In current literature, sex and sexuality education is defined differently. However, sexuality education focuses on developing and strengthening the ability of children and young people to make conscious, satisfying, healthy and respectful choices regarding relationships, sexuality and emotional and physical health [20]. In Ghana, the term sexual and reproductive health education is widely employed in both policy and programmes [20], and we therefore adopted that terminology in this paper. By sexual and reproductive health education, reference is made to both formal (e.g. school-based) and informal education on such issues as puberty/physical changes in the body; reproductive organs; menstruation and menstrual hygiene; pregnancy and childbirth, HIV/AIDS; other STIs; where to access STI/HIV services; contraceptive methods, including where to get contraceptive methods and how to use contraceptive methods; appropriate sexual behaviour; abstinence/chastity; moral issues related to sexuality, and sexual and reproductive rights. In the context of Ghana, the aims of sexual and reproductive health education include helping people to acquire accurate information about human sexuality, sexual and reproductive health, explore and nurture positive values and attitudes towards their sexual and reproductive health, and develop self-esteem, respect for human rights and gender equality, and develop skills that encourage critical thinking, communication and negotiation, decision-making and assertiveness as regards sexual and reproductive health matters [20].

Although sexual and reproductive health education is just one component in a multifaceted approach to improve sexual and reproductive health outcomes, it could provide a structured opportunity for girls/women, especially in urban slums, to gain knowledge and skills, to explore their attitudes and values, and to practice decision making and other life skills necessary for making healthy informed choices about their sexual and reproductive health [20]. For instance, timely provision of accurate and comprehensive information and life skills training regarding sexual and reproductive health and rights is essential for adolescents to achieve sexual health and rights and avoid negative health outcomes [20]. Similarly, sex education may introduce people to a wide array of reproductive health issues such as education on sexually transmitted infections (STIs), types and sources of contraception as well as their merits and demerits [21]. Sex education may also improve contraceptive knowledge, increase chances of both contraception use at first intercourse and contraception use in a lifetime, and effective usage of current method [22–24].

Like many countries in Africa, however, public conversation about sex and sex education and sexuality has historically been deemed taboo subjects [25]. This has resulted in very limited parent–child communication and education about sex, sexuality and sexaul and reproductive health [25]. The situation is however beginning to change partly because Ghana has agreed to several international declarations (e.g., the Abuja and Maputo Declarations) that have informed governmental decisions and actions on sexual and reproductive health (SRH), including specific changes relating to improving access to services and information for adolescents. While Ghana is yet to develop and/ or implement a comprehensive sexuality education policy, at the national level, there is currently a legal framework as well as a supportive policy environment for the development and implementation of SRH education in Ghana [20]. In 2000 for example, the government published its first *Adolescent Reproductive Health Policy* (ARHP), which adopted a multi-sectoral approach to addressing adolescent reproductive health issues [20]. The ARHP explicitly encouraged and led to the inclusion of a sexual and reproductive health education component in the educational curriculum at the primary, junior high and senior high school levels [20]. In 2013, Ghana’s *National HIV/AIDS and STI Policy* further advocated the inclusion of age-appropriate SRH education in the school curriculum, which includes lessons on HIV/AIDS and other STIs. In this regard, the Ministry of Education and the Ghana Education Service have collaborated with key agencies, including the Ministry of Health and the Ghana Health Service, to provide sex, sexuality and reproductive health education in schools. Although a range of topics are included in the educational curricula, a recent study noted that the topics are often limited in scope—there is a major focus on abstinence and, in some cases, a fear-based or negative perspective on sexuality [20]. This school-based sexual and reproductive health education is also only limited to young people aged 8–19 [20]. Besides, it is not clear to what extent efforts to provide sexual and reproductive health education in schools are reaching young girls and women in urban slums who may either be out-of-school or have not had any education at all. It has been estimated that about 37.9% (5.4 million) of urban residents in Ghana live in slums [26]. Most of these slums are in Accra, the capital city of Ghana, and face the most difficulty accessing sexual and reproductive healthcare information and services [27].

Previous studies on sex, sexuality and reproductive health education have investigated the proportion of school going population that have received sex education [28, 29]. However, to date, our extensive search indicated that no scholarly evidence exists on the association between sexual and reproductive health education and contraception use among the ever-increasing urban slums in Ghana. As a result, this study sets out to test the hypothesis that Accra slum women and girls who have had sexual and reproductive health education have higher chances of ever using contraception. It is anticipated that this study will ignite policy dialogue on tailored approaches by which sexual and reproductive health education can be utilized to champion contraceptive use among urban slum residents of Accra and eventually contribute substantially toward achievement of SGD 5.

## Methods

### Study design

A cross-sectional quantitative survey was conducted as part of a larger mixed methods study that aimed to identifying and develop low-cost and acceptable family planning interventions and service delivery models for urban informal settlements in Accra, Ghana.

### Study area and population

The study was conducted in two slums (i.e. Agbogbloshie and Old Fadama) in Accra, Ghana. Agbogbloshie and Old Fadama cover about 31.3 hectares of land [30]. Agbogbloshie and Old Fadama are both heavily populated and resource-poor environments. Although the exact population of Agbogbloshie is not easy to determine, the 2010 Population and Housing Census reported that 8305 (54% female and 46% male) people lived in Agbogbloshie [31]. Old Fadama however has much highe population: approximately 100,000 people in 2018 [31, 32]. The majority of people in Old Fadama and Agbogbloshie work in the informal sector. The two settings were considered as study sites because they are much larger in terms of land area and population than other slums. Because of the relatively large population size, there were enough potential respondents for this study's recruitment. They were also chosen because, unlike nearby areas such as Ussher Town and James Town, which are mostly populated by Ga people, Agbobloshie and Old Fadama residents come from all over Ghana and represent diverse ethnic groups [30]. This diversity also helped to ensure that diverse views are represented in this study. The study’s primary respondents comprised female slum dwellers aged 15–49.

### Sample size determination

Cochran’s [33] sample size determination formula for cross-sectional studies was used to estimate the sample size for the study**.** In estimating the sample size, the following assumptions were made: (a) Confidence level is assumed to be 95%, (b) Margin of error is assumed to be 5% (i.e. = 0.05), (c) Based on modern contraceptive use prevalence of 21% in the Greater Accra region as reported in the recent Ghana Maternal Health Survey [34], it was assumed that 21% of the girls/women that will be surveyed in this study will be modern contraceptive users. Based on these assumptions and using 80% study power, the minimum sample size required to detect statistical association in the study was estimated to be 503. To account for non-response and also ensure that the study is sufficiently powered, a 100% upward adjustment (i.e. 100/100 × 503 = 503) was made to the minimum sample size estimated above. The final estimated sample size was therefore 1006 (i.e. 503 + 503). However, in this paper, a sample size of 691, made up of participants who provided responses to the question on their ever use of contraception, sex education as well as those with complete information on all the other variables of interest, was used.

### Sampling and recruitment of participants

Because the settlements in Agbogbloshie and Old Fadama are relatively unorganized, a convenience sampling technique was used to select respondents for the study. A number of steps were involved in contacting and recruiting sexually active women and girls for the study. First, using appropriate community engagement strategies, the study was presented to community leaders. Second, the study was advertised and suitable participants were recruited through formal and informal social networks (e.g., youth groups and women's groups). Lastly, sexually active women/girls aged 15–49 were asked to register with one of three local community recruitment officers stationed in Agbogbloshie and Old Fadama, or call a dedicated phone number operated by a qualified research assistant stationed at the Principal investigators’ institution. All women/girls who approached the qualified research assistant or the community recruitment officers were screened individually for eligibility.

### Data collection methods and tools

A structured questionnaire was designed, pre-tested and used to collect the data. The questionnaire was used to gather information on contraceptive use and other socio-demographic, reproductive/maternal and behavioural characteristics of participants. Most of the questions were adapted from the Ghana Demographic and Health Survey questionnaire and the Ghana Maternal Health Survey. After designing the questionnaire, it was imported into the REDCap (Research Electronic Data Capture) platform (see https://projectredcap.org/software/)—a software used to design and collect electronic data using electronic gadgets such as mobile phones or tablet computers. This software allowed data to be collected and saved automatically on the device, as well as uploaded to an online server via the internet. This technology helps to reduce the risk of data loss, which is common in paper-based questionnaires that are easily destroyed by natural events such as rain. Similarly, the automatic data saving eliminated the need for a separate data entry phase, which is common with paper-based questionnaires. The built-in skip logics also reduced the possibility of missing data. In all, eight (8) graduate research assistants were trained to collect the data face-to-face using one of four languages—English, *Twi, Ga* and *Hausa*. COVID-19 protocols were strictly adhered to, including maintaining appropriate social distance of 1–2 m when interacting with participants, compulsory wearing of nose/face mask for both the data collection team and participants, and regular hand washing and use of alcohol-based hand sanitizers. Participants were not paid for their participation in the study. Rather, each participant received a token gift of one cake of Geisha soap and one disposable nose/face mask after the interviews.

### Derivation of study variables

#### Outcome variable

The outcome variable for this study was ever use of contraception. It was derived from the question, ‘have you ever used any contraceptive?’ The responses to this question were “yes” and “no”. Respondents who chose “yes” as a response were asked a follow-up question (Which method have you ever used?). For this question, several responses were obtained including female sterilization, male sterilization, Intrauterine device (IUD), injectable, implants, pill, male condom, female condom, lactational amenorrhea method (LAM), emergency contraception and other modern method, rhythm/calendar method, withdrawal, and other traditional method.

### Independent and control variables

The key independent variable for the study was sexual and reproductive health education. Specifically, the variable was derived from the question “Have you received any form of education about sex, sexuality and reproductive health at any point in your life? And the responses were “Yes” or “No”. Sexual and reproductive health education was operationally defined in line with Ghana’s current sexual and reproductive health education policy framework as any formal (e.g. school-based) and informal education on such issues as puberty/physical changes in the body, reproductive organs, menstruation and menstrual hygiene, pregnancy prevention, HIV/AIDS and STIs prevention and treatment, contraceptive use, appropriate sexual behaviour, abstinence/chastity and moral issues related to sexuality, and sexual and reproductive rights aimed at developing and strengthening the ability of individuals to make conscious, satisfying, healthy and respectful choices regarding their sexual and reproductive health [20]. We did not assess the comprehensiveness of the sexual and reproductive health education. Rather, we focused on whether respondents have ever received any form of formal or informal education on sex, sexuality and reproductive health as outlined above. During data collection, research assistants took time to explain and clarify the concept of sexual and reproductive health education to the respondents, including providing examples of the different aspects of sexual and reproductive education that we intended to measure. This ensured that all respondents had the same or similar understanding of what was being asked and measured.

Apart from sex education, six control variables were considered in the study. These comprised age (15–24, 25–34, 35–49), level of education (no formal education, primary, secondary/higher), ethnicity (Akan, Ga-Dangme, Ewe, Mole-Dagbani, Gurma, and other), exposure to radio (Yes, No), marital status (never married, married, cohabiting, and separated/divorced/widowed) and national health insurance scheme (NHIS) subscription (Yes, No). These variables were chosen based on their practical significance and their theoretical and empirical relevance to ever use of contraception [9, 12, 16, 29].

### Data processing and analyses

All data files were exported into Stata version 14.2 for cleaning, coding and possible recoding and analysis. Data cleaning was done by identifying outliers/anomalies and checking for consistency among and across variables. Frequency distributions and cross tabulations were specifically run to aid the data cleaning process. Descriptive and inferential analysis were then conducted. At the descriptive level, frequencies and percentages were used to present information on demographic characteristics and the prevalence of ever use of contraception (see Table 1). At the inferential level, two binary logistic regression models were fitted (see Table 2). This analytical approach was the most suitable option because of the fact that our dependent variable, ever use of contraception, was a binary variable (Yes/No). The first model (Model I) accounted for sexual and reproductive health education and ever use of contraception. In model II, we adjusted for the effect of sex education and other socio-demographic characteristics. The results for Model I and Model II were presented as crude odds ratios (cOR) and adjusted odds ratios (aOR) respectively. In all the analysis, statistical significance was set at p-value less than 0.05.

**Table 1:** Prevalence of ever use of contraceptives among women in slums, Accra

Variable	Frequency	Percentage	Ever used contraception
Ever used contraception			77.28
Sex education			
Yes	299	43.27	81.61
No	392	56.73	73.98
Age			
15–24	362	52.39	76.24
25–34	207	29.96	79.71
35–49	122	17.66	76.23
Education			
No formal education	189	27.35	76.19
Primary	115	16.64	85.22
Secondary/higher	387	56.01	75.45
Marital status			
Never married	388	56.15	78.35
Married	198	28.65	72.22
Cohabiting	40	5.79	90.00
Separated/divorced/widowed	65	9.41	78.46
Ethnicity			
Akan	160	23.15	80.62
Ga-Dangme	80	11.58	76.25
Ewe	90	13.02	85.56
Mole-Dagbani	285	41.24	73.68
Gurma	16	2.32	68.75
Other	60	8.68	76.67
Exposure to radio			
Yes	543	78.58	80.48
No	148	21.42	65.54
NHIS subscription			
Yes	580	83.94	78.62
No	111	16.06	70.27
N	691	691

**Table 2:** Sexual and reproductive health education and ever use﻿ of contraceptive among women in slums in Accra, Ghana

Variable	Model I cOR [95% CI]	Model II aOR [95% CI]
Had Sexual and reproductive health education		
Yes	Ref	Ref
No	0.641* [0.443, 0.928]	0.652* [0.436, 0.975]
Age		
15–24		Ref
25–34		1.325 [0.826, 2.127]
35–49		1.068 [0.552, 2.066]
Education		
No formal education		Ref
Primary		1.507 [0.793, 2.863]
Secondary/higher		0.687 [0.448, 1.054]
Marital status		
Never married		Ref
Married		0.577* [0.353, 0.944]
Cohabiting		2.786 [0.876, 8.861]
Separated/divorced/widowed		0.91 [0.416, 1.992]
Ethnicity		
Akan		Ref
Ga-Dangme		0.801 [0.412, 1.558]
Ewe		1.388 [0.679, 2.837]
Mole-Dagbani		0.779 [0.472, 1.288]
Gurma		0.655 [0.219, 1.961]
Other		0.945 [0.434, 2.056]
Exposure to radio		
Yes		2.183*** [1.379, 3.456]
No		Ref
NHIS subscription		
Yes		Ref
No		0.626 [0.385, 1.018]
N	691	691
Pseudo R^2^	0.008	0.06

Exponentiated coefficients; 95% confidence intervals in brackets; cOR, crude Odds Ratio; aOR, adjusted Odds Ratios; CI, Confidence Interval; Ref, reference category

^*^p < 0.05, ^**^ *p* < 0.01, ^***^ *p* < 0.001

## Results

### Descriptive findings

From Table 1, approximately 53% of the respondents were aged 15–24. More than half of them had secondary/higher level of education (56.01%) and were never married (56.01%). The majority of them belonged to the Mole-Dagbani ethnic group (41.24%). Majority of them were exposed to radio (78.58%). The greater percentage (83.94%) of the women had subscribed to the NHIS. More than half (56.73%) of the women had never received sexual and reproductive health education. However, 81.61% of those who had received some form of sexual and reproductive health education ever used contraception. More than 76% of women of all age categories had ever used contraception and this was highest (79.71%) among those aged 25–34. The highest proportion of ever used contraception was among cohabiting women (90.00%), Ewe women (85.56%), those with primary education (85.22%), those exposed to radio (80.48%), and those who have subscribed to NHIS (78.62%).

### Types of contraceptive ever used

**Fig. 1:**
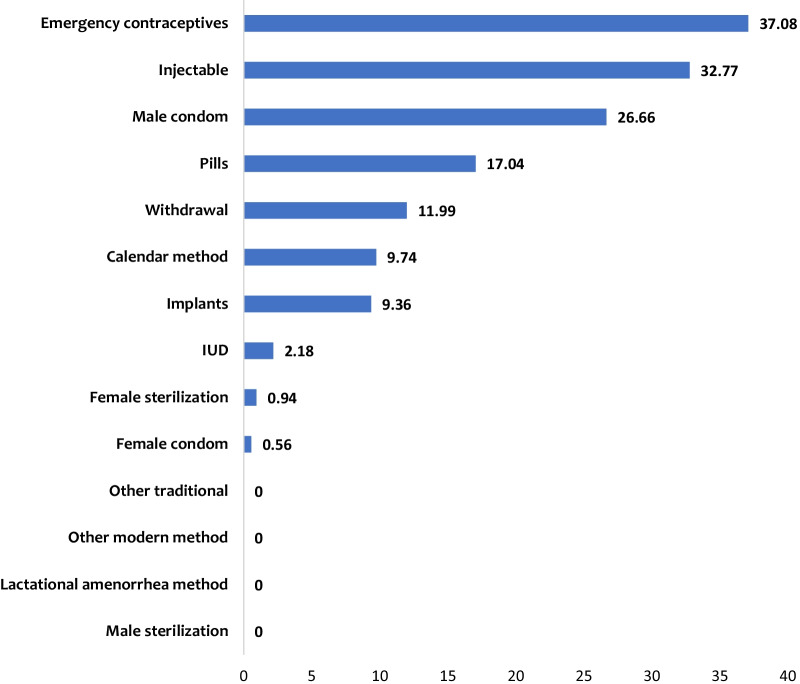
Types of contraceptives ever used

Among the 77.28% of the respondents who have ever used contraceptives (Table 1), the predominant contraceptives ever used were emergency contraceptive (37.02%), injectables (32.77%), male condom (26.66%) and pills (17.04%). The least methods ever used were female condom (0.56%) and female sterilization (0.94%) (Fig. 1).

### Inferential results

#### Binary logistic regression analysis on sex education and ever use of contraception

As shown in Table 2, women who had not received any form sexual and reproductive health education had lower odds of ever using contraception (OR = 0.641, 95% CI 0.443, 0.928] and this persisted after controlling for the effect of demographic factors (AOR = 0.652, 95% CI 0.436, 0.975] compared to those who have ever received some form of sex education. With the control variables, married women (AOR = 0.577, 95% CI 0.353, 0.944] had lower odds of ever using contraception compared to never married women. However, women exposed to radio had higher odds (AOR = 2.183, 95% CI 1.379, 3.456] of ever using contraception compared with those who are not exposed to radio (Table 2).

## Discussion

In this study, we tested the hypothesis that women living in urban slums in Accra who have had some sexual education have higher chances of ever using contraception. To find answers to this hypothesis, we determined the proportion of women who had sex education and those who had ever used contraceptives. In terms of the prevalence of sex education and contraceptive use, we found that less than half of the women in urban slums ﻿in Accra had sex education but most of them had ever used contraceptives. Further analysis supported our hypothesis that urban slum women in Accra who had no sex education were less likely to use contraceptives compared to those who had sex education. Being single (not married) and exposure to radio also statistically significantly increased the odds of ever use of contraceptives.

We found a low prevalence of sex education among women in urban slums in Ghana. The low prevalence of sex education identified in our study supports studies that have also found low prevalence of sex education in Ghana [20, 35, 36]. The possible reasons for the low prevalence of sex education in the current study could be related to socio-cultural norms around sex education in Ghana and the misconceptions around sex education that exist in the country [33, 34]. For example, there is a common misconception that sex education predisposes young people to early sexual activity. Again, sex education is considered as contradicting existing socio-cultural norms and religious beliefs that emphasizes abstinence [37, 38]. Despite the similarities in the prevalence of sex education in the current study and previous studies, there exist differences in terms of the target population and data source for these studies. Notwithstanding, the findings of our study reflect the current status of sex education in Ghana and calls for continuous efforts to make sex education available to Ghanaians outside of the formal school system, especially for those who live in disadvantaged geographical locations like slums.

Despite the low prevalence of sex education, we found a relatively high prevalence of ever use of contraception, with majority of the women using emergency contraceptives. Similar but comparatively lower prevalence of contraceptive use among women in urban slums have been found in studies conducted in other low-and middle-income countries, including Bangladesh [18, 39] and Kenya [11, 14]. It is not entirely clear why a higher proportion of the women/girls sampled were contraceptive users, especially emergency contraceptives. However, a number of reasons could be adduced. Many urban slums in Accra are inhabited by commercial sex workers who have been found to engage in high risk sexual behaviours, including unplanned sex and multiple sexual partners [40–42]. These populations also often have weaker bargaining power to demand safe sex. Emergency contraceptive use may therefore be a direct response to both unplanned and unsafe sex among this population in order to prevent unwanted pregnancy. As a result, recent interventions by Ghana’s AIDS Commission in partnership with the Ghana Health Service and other partners have focused on provision of contraceptive commodities (e.g. condoms and lubricants) to commercial sex workers as special populations [20, 25]. We believe this could have contributed to the relatively high prevalence of contraceptive use in our sample. It is also possible that the convenient sampling strategy we adopted could have led to self-selection of more contraceptive users into our study. We have acknowledged this in our study’s limitation section as a potential selection bias and a limitation of our study. Notwithstanding these explanations, the high prevalence of contraceptive use in general and specific use of emergency contraception calls for further research to understand the underlying reasons for these observations.

In terms of the association between sex education and contraceptive use, we found that women who had no form of sex education were less likely to ever use contraceptives compared to those who had sex education. This is not surprising given that numerous studies have concluded that sex education potentially increases rates of contraceptive use at first sexual intercourse [22, 24, 43, 44]. Studies have further shown that sex education introduces people to a wide range of information on reproductive health issues such as education on STIs, types and sources of contraception as well as their merits and demerits [21]. Generally, sex education often enhances contraceptive literacy, raises the likelihood of using contraception at any point in one's life, as well as effective and consistent usage [22]. As a result, slum residents who have had any form of sex education are more likely to utilize contraception. This relationship, however, is strongest for those exposed to media, but lower among those who are married as found in the current study. To enhance contraceptive use, there is a need to intensify sexual and reproductive health education among women in urban slums, taking into consideration differences in contraceptive use according to their marital status and media exposure.

## Limitations of the study

The findings of this study should be interpreted with certain limitations in mind. The study adopted a cross-sectional design. Consequently, causal inference cannot be made between sex education and ever use of contraception. Again, since the study used a snowball recruitment strategy based on waiting for women and girls to register themselves, there is the possibility of under sampling of some of the women who may have been, for example, less interested in the study, or more nervous about the topic due to personal circumstances, or less educated all of which may bias the results. In addition, the questions were asked orally by study team members for the respondents to respond. This might have also introduced some social desirability biases. Notwithstanding, the study presents a true account of the relationship between sex education and contraceptive use in urban slums of Accra.

## Conclusion

The study revealed a low prevalence of sex education and high prevalence of ever use of contraception among women in urban slums in Accra. Sex education was found to increase the prevalence of ever use of contraceptive. These findings call for more and targeted sexual and reproductive health education among reproductive aged girls and women in urban slums in Accra using existing informal social networks and local media platforms. Such education should be done taking into consideration differences in contraceptive use according to their marital status and media exposure.

## Data Availability

All relevant data are included in this paper.

## Abbreviations

aOR: Adjusted Odds Ratio
CI: Confidence Interval
cOR: Crude Odds Ratios
IUD: Intrauterine device
LAM: Lactational amenorrhea method
SDG: Sustainable Development Goal
SRH: Sexual and reproductive health
